# Abnormal electrocardiogram T wave pattern in ischaemic stroke

**DOI:** 10.1007/s12471-022-01690-y

**Published:** 2022-05-03

**Authors:** C. Soares, M. Temtem, A. Sá, R. Rodrigues

**Affiliations:** 1 Centro de Saúde do Bom Jesus, ACES RAM, Funchal, Portugal; 2 Cardiology Department, Hospital Dr. Nélio Mendonça, Funchal, Portugal

**Fig. 1:**
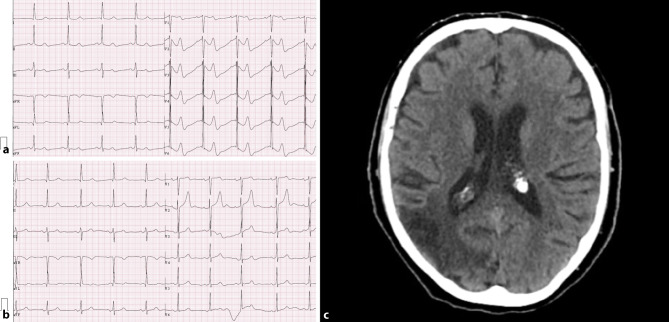
Electrocardiogram at **a** primary care emergency services and **b** hospital admission. **c** CT scan, at hospital admission, showing ischaemic area in right temporal-occipital-parietal cortex

An 88-year-old female with a history of hypertension, dyslipidaemia and hypothyroidism but without known pre-existing heart disease arrived at the primary care emergency services with sudden confusion and agitation. On admission, electrocardiography (ECG) showed sinus rhythm (SR) with bizarre and distorted T waves associated with a prolonged QTc interval (589 ms) in leads V2–V6 (Fig. 1a). She was transferred to hospital, and ECG was repeated 2 h later, which showed a different pattern: SR and prolonged QTc interval without the previous ST-T changes (Fig. 1b). Cerebral computed tomography (CT) imaging identified an ischaemic area in the right temporal-occipital-parietal cortex (Fig. 1c). The patient was discharged after 7 days, with moderate mental confusion.

Ischaemic strokes may be associated with ECG changes such as prolonged QT interval, ST-segment depression, T wave inversion or abnormal U waves. The precise mechanism has not yet been identified, but changes are usually transient [1]. There is a lack of evidence for prehospital ECG changes in acute stroke patients [2]. Recognition of this unusual pattern may point to a differential non-cardiac diagnosis and prevent delay in clinical decision-making and early treatment.
